# Chances and risks of publication of quality data – the perspectives of Swiss physicians and nurses

**DOI:** 10.1186/1472-6963-12-368

**Published:** 2012-10-26

**Authors:** Regula Heller, David Schwappach

**Affiliations:** 1 University of Zurich, Zurich, Switzerland; 2 Scientific Head – Swiss Patient Safety Foundation, Zurich, Switzerland; 3 Institute for Social and preventive Medicine (ISPM), University of Bern, Bern, Switzerland

## Abstract

**Background:**

The release of quality data from acute care hospitals to the general public is based on the aim to inform the public, to provide transparency and to foster quality-based competition among providers. Due to the expected mechanisms of action and possibly the adverse consequences of public quality comparison, it is a controversial topic. The perspective of physicians and nurses is of particular importance in this context. They are mainly responsible for the collection of quality-control data, and are directly confronted with the results of public comparison. The research focus of this qualitative study was to discover what the views and opinions of the Swiss physicians and nurses were regarding these issues. It was investigated as to how the two professional groups appraised the opportunities as well as the risks of the release of quality data in Switzerland.

**Methods:**

A qualitative approach was chosen to answer the research question. For data collection, four focus groups were conducted with physicians and nurses who were employed in Swiss acute care hospitals. Qualitative content analysis was applied to the data.

**Results:**

The results revealed that both occupational groups had a very critical and negative attitude regarding the recent developments. The perceived risks were dominating their view. In summary, their main concerns were: the reduction of complexity, the one-sided focus on measurable quality variables, risk selection, the threat of data manipulation and the abuse of published information by the media. An additional concern was that the impression is given that the complex construct of quality can be reduced to a few key figures, and it that it is constructed from a false message which then influences society and politics. This critical attitude is associated with the different value system and the professional self-concept that both physicians and nurses have, in comparison to the underlying principles of a market-based economy and the economic orientation of health care business.

**Conclusions:**

The critical and negative attitude of Swiss physicians and nurses must, under all conditions, be heeded to and investigated regarding its impact on work motivation and identification with the profession. At the same time, the two professional groups are obligated to reflect upon their critical attitude and take a proactive role in the development of appropriate quality indicators for the publication of quality data in Switzerland.

## Background

The trend of allowing quality and performance data from medical and nursing services in acute-care hospitals to be accessible to the general public has risen sharply in recent years. In the United States and Great Britain, the publication of comparative quality data has been practiced in various forms for quite some time (e.g. rankings, report cards, provider profiles and consumer reports). In the USA it was established in the beginning of the nineties, and in Europe the first initiatives originated in Scotland in 1994 [1].

This development is just beginning in Switzerland. In 2009 the publication of mortality rates, which were based on routine data from Swiss university hospitals, and then were released by the Swiss Federal Office of Public Health, triggered large and critical discussions [2]. In Switzerland, the discussion about the publication of quality data has only been concrete for a few years. The Swiss health system is decentralized and federal. The highest level of authority for health care delivery lies with each canton. This decentralized organization of the health care system has many advantages, but it can, in certain areas, prevent reforms and improvements. This Swiss health care culture is noted for giving a lot of autonomy to individual cantons with minimal government regulations, especially in regards to quality control and transparency.

The release of quality data is based on several of the following claims. The publication of performance quality data would achieve transparency. Providers with good quality of care would receive confirmation for their work. Those providing poor quality services could be offered an incentive to conduct quality enhancing measures. Citizens pay the taxes and premiums and are also potential patients; therefore, they should have the comparative information available to them. This data would then assist them in their decision of which hospital to choose. As a result of this quality oriented selection of providers by patients, and changes in the referral patterns by physicians, shifts in service volumes and market shares between “good” and “bad” providers are expected. This quality performance competition would especially be valued by financers, health insurance companies and health directorates.

However, findings from the literature show that these mechanisms of action are not expected to occur to such a large degree. The effects of quality data being published was examined in several studies from the United States and Great Britain. Several systematic reviews and articles address these questions [3-8]. There is a discrepancy between the importance that the general population would attach to the published quality data, and the consequences that the consumers and patients would experience from the data. There is little evidence that the patients’ and referring physicians’ hospital choice will be affected due to the publication of quality comparisons. The expected selection of providers, and thereby the induced quality competition seems to not have occurred as yet. The following are mentioned as reasons: personal references, personal experience, and that reputation and recommendation of an institution from the family doctor would be more heavily weighed than published data [3,4,9-11].

Patients’ lack of interest in published quality data and hence the limited benefits, can be seen as a consequence due to the way the information is offered. They are often designed too much from the perspective of the stakeholders, and too little from the perspective of the users [12]. An analysis of quality reports from Germany confirm that they are comprehensible only to patients who have above-average reading and language skills [13].

In the above mentioned studies, some negative ramifications of publication of the data were described, such as selection by hospitals based on risk or a very one-sided view on the easily measurable aspects of quality (in relation to those less accessible to measurement). This one-sided view of placing significance only on what is measurable, also known as “tunnel vision”, increases the risk that the priorities regarding quality improvement would be based on easily measurable areas, and that other more important areas may be potentially neglected [14,15]. Also, it can be assumed that the use of quality data for selection of the hospital is highly correlated with education (health literacy) and other socio-economic factors, and thus effects of inequality in health care among population groups are feared.

In the development of appropriate concepts and quality indicators for publication of quality data, the following groups are represented: politics, health insurance, economics, health economics, patient organizations and hospital directorates. The two largest professional groups in health care, the physicians and nurses employed in hospitals, have a very minimal role in this discussion. The few studies that have examined the perspectives of hospital directorates, chief physicians and nursing administrators in regards to public rating systems and quality comparisons, indicate that they have a rather critical attitude. Inadequate presentation of performance, lack of confidence in data quality and the risk of adverse consequences are mentioned as reasons [14,16]. The opinions, perspectives and evaluations provided by nurses and physicians are significant, and given the size of these two professional groups, it is essential not to underestimate their influence on further development of this issue.

The research focus of this qualitative study was to discover what the views and opinions of the Swiss physicians and nurses were regarding these issues. It was investigated as to how the two professional groups appraised the opportunities as well as the risks of the publication of quality data in Switzerland. This work may provide clues as to what the impact of publication of quality data would be for the affected employees, and what accompanying measures might be necessary to take in order to counter-act the negative effects.

## Methods

### Design

To guide the research, an explorative, qualitative approach was chosen. This approach seems suitable for the issue, because in Switzerland knowledge regarding this topic is lacking.

### Data collection

Collection of data was conducted via four focus groups from Swiss acute care hospitals. Two focus groups consisted of physicians and two groups consisted of nurses. All physicians and nurses were employed in Swiss acute care hospitals and the location and size of the hospitals varied. Some participants worked in a university hospital, some in city centre hospitals and others in regional hospitals. The hospitals were located in four different cantons in the German speaking regions of Switzerland. The discussion groups were assembled in accordance to the principles of Theoretical Sampling. Therefore, the sample groups were assembled and adapted to the research question, with both genders present and all containing typical representatives of the target group [17].

To recruit the participants, various forms of inquiry were utilized. Several people were requested to participate directly by e-mail. Individuals known professionally by the first author, as well as other individuals whose names were provided from a third party (such as quality control representatives from these hospitals), were also recruited. Other participants were recruited via an information letter regarding the objectives and content of the focus groups. This information was distributed within hospitals via contact persons. Interested participants notified the author directly.

Participants received a written statement informing them about the aims of the study and the guaranteed confidentiality of the data. At the beginning of each focus group, participants were required to give written consent for their voluntary participation, for the digital recording of the discussions and for the upholding of the confidentiality of the information obtained from the other participants. Ethical approval is not required for this type of study in Switzerland. A guide was developed regarding the topic of interest as well as the activities of the group discussion [17]. It was designed to be very open, in order to allow for as much free access to the subject as possible and in order not to limit the diversity of ideas. The following topics were discussed:

Relevance of the theme “publication of quality data” for the professional groups.

Quality of performance would be public – what effects may be triggered with Swiss physicians and nurses?

Impact of public benchmarking on motivation, work climate, identification with the organization, etc. in Switzerland?

Competition and rivalry – the meaning of and the assessment of these two aspects.

Assessment and evaluation of the movement towards public reporting in Switzerland.

In order to facilitate discussion and to approach to the subject/topic, the necessary background knowledge and current developments regarding the publication of quality data were provided. Hypothetical scenarios were then discussed in regards to quality data in order to stimulate discussion. The discussions were conducted with a trained moderator and the author was present as an observer. The contents of the discussions were digitally recorded and then professionally transcribed.

### Data analysis

The extensive text material was systematically analyzed by means of a summative, qualitative content analysis using the computer program MAXQDA [18]. Categories were extracted using an inductive approach. Data analysis was conducted separately for each occupational group, because it was assumed that there would be differences between the two professional groups regarding their professional socialization, communication of their culture and their occupational and public health perspectives.

For the first step, statements in the text material that revealed a relationship to the research question were identified and coded. In other words, statements were assigned to the chosen thematic concepts (codes). The newly developed groups, in a further step, were combined into super ordinate categories. These super ordinate categories were then merged into one category system. After this, in order to test the developed category system, a person not involved in the analysis process compared the contents of the categories with the original material. Finally, individuals from the focus groups reviewed and analyzed the results. With these two latter steps, the reproducibility and consistency of the category system was validated. For presentation of the results, representative quotes were selected.

## Results

### Description of sample

In total, 15 nurses and 8 physicians employed in Swiss acute care hospitals were recruited for the focus groups. The age and work experience of the two groups varied considerably (see Table 1). The focus group discussions lasted between 70 and 85 minutes.

**Table 1:** Age, work experience, position and professional functions

	**Nurses**	**Physicians**		
Age (years)	25 - 59	38 - 59		
Work experience (years)	1 - 37	12 - 34		
Employment in hospital (years)	1 - 16	0.5 - 19		
		
Professional role	Nurse	7	Senior physician	1	
	Care expert	4	Senior consultant	5	
	Station manager	5	Chief physician	2	
	Leader – nursing development	1			

### Description of results

The participants described in detail the opportunities and risks of the publication of quality data. They gave insight into their perspectives and attitudes regarding how they perceived the recent development of the public reporting of quality data in Switzerland, and which reactions were triggered within them due to this. In the data four main categories were identified. The themes that emerged from the categories were mentioned by both professional groups, although the emphasis was different. The following results are for both groups with their specific differences being noted:

a) **Appraisals and reactions to the recent development of the public reporting of quality data in Switzerland.**

b) **What is quality and what is portrayed in the publication of this information?**

c) **Opportunities associated with the publication of quality related information.**

d) **Risks associated with the publication of quality related information.**

### Appraisals and reactions to the recent development of the public reporting of quality data in Switzerland

The publication of quality data is gauged as social movement that will be observed not only in the health care system. The trend towards having easily accessible and readily available knowledge without any accompanying in-depth background information is a reflection of the current information age. The relevance of this development is assessed differently by nurses. Some nurses are hardly confronted with it or they view themselves as not being the producers of the published results. However, data that has already been published, for example via internet comparison services, may raise concerns and discussions. There are varying perspectives depending upon the purpose and intent in which quality data is published. Publications in newspapers, radio and television are critically evaluated.

"“If it is a journalist, who’s only looking for a good headline, it makes me angrier than if it is a noble attempt to find out differences with factual questions. In the latter case, there are also professional interests behind it. It depends on how it is done in order to get better numbers”."

A publication in a professional journal is rated more positively. However, depending upon, the publishing physician’s experience, comparative quality data often lacks a specific research question or hypothesis. Due to this, recent publications such as the mortality rates from the Swiss Federal Office of Public Health are rated as untrustworthy and destructive.

"“It is annoying, that unsuitable information sometimes gets out. It is then being severely criticized, without checking whether it has any statistical significance. Certain things or hospitals are being shot down, before one thinks about if all that is said is true. It is annoying, because it is unfair and because it is sometimes stupidity.”"

In particular, the physicians explain the development towards the public reporting of quality data, as a response to the failure of politicians in dealing with grievances. Due to parliamentary and societal pressure, the Swiss Federal Office of Public Health, from the perspective of physicians, reacted inadequately and ineffectively to this development. The increasing commercialization of health care associated with this recent trend, is also noticeable to the nurses and physicians. The participants of study are critical as to how the principles of a free market economy, such as the competitive aspect, are thoughtlessly applied to health care.

For both professional groups, the collection of quality data and the associated measures to improve quality are indispensable and are a part of clinical practice. The latest developments are therefore regarded with mistrust and viewed as being discrediting to past performance. Moreover, it is also perceived as threatening, should it have an influence on the distribution of future resources.

### What is quality and what is portrayed in the publication of this information?

Both professions see a big problem in the obscurity of the definition of quality. In the context of publications, careless use of the term quality is seen as discrediting. From the perspective of physicians, the complexity of the concept of quality is often underestimated by many people in the health care sector, and it is therefore used very indiscriminately. In addition, by reducing quality to individual figures, only partial aspects of the care process in hospitals are depicted. Core competencies as well as economic aspects could be overlooked. Among the understood core competencies are characteristics such the departments’ regulations, respecting the patients’ will and bringing the patient into an improved state of health. The ambiguity of the definition makes it difficult, even within the professional groups themselves, to identify the relevant and appropriate indicators. For example, within the two occupational groups, it was controversial if patient satisfaction was a relevant and appropriate indicator of quality of care.

### Opportunities associated with the publication of quality related information

The few statements of the participants that pointed to the possible opportunities associated with the publication reflected only “precursors” to potentially positive effects. For most participants, the benefits of obtaining quality data for internal purposes are undisputed and the associated potential for quality improvement is recognized.

"“I consider it a great opportunity within the professional circle, and also for getting help or gathering ideas. However, I do not find it necessary for all of humanity to know about it.”"

A potential benefit attested to, in particular by the nurses, is that with the publication of quality data, the comparability of the data is ensured and the necessary background information is provided (under the condition that data are collected correctly). Negative speculations are nurtured if data is collected and then kept confidential. A proactive position for the hospitals would be to have their data gathered by specialists and then communicated to the public. This could be an alternative to speculative and potentially damaging headlines in the media. For some nurses a competition is actually appreciated, and may have positive aspects. Competition does have negative components, however it is also positive, as it can be motivating and businesses and health care can develop from it.

### Risks associated with the publication of quality related information

#### Publication represents reduction of complex circumstances

Both professional groups have little trust in the quality of the data that is collected, and evaluate the data as undifferentiated and too narrow in focus. A publication is unavoidably a reduction of complex circumstances to single figures. Therefore, from the perspective of the two professional groups, comparisons are very problematic and open the door to misinterpretations and the drawing of inaccurate conclusions. Such a development is viewed by the two occupational groups as being counterproductive and potentially damaging. Moreover, for the participants, it would be seen as discrediting and demotivating if they would be submitted to such negative consequences.

#### Incentives for risk selection

The publication of quality data creates not only false incentives for risk selection and “nit-picking” within private hospitals or hospitals with fewer service contracts, but also sends the wrong message to the general public.

"“I think it would be similar to nit-picking. One might think that it would be better to not go at all to any hospital, for it would certainly turn out badly. This could then have negative consequences for the health of the individual.”"

Particularly with the publication of mortality rates, the population receives the wrong message. For example, if it is the hospitals’ goal to achieve as low as possible mortality rates, then there is the risk of a poorer quality of life and there would be financial and sociopolitical consequences to consider. Hospitals that support patients in their dying process would have a higher rate than hospitals that re-locate the dying patient as quickly as possible. Such complex issues would be hidden through the reduction in the figures, and would therefore be neither evident nor assessable to the general public.

The construct of the publication of data is based on the assumption that patients have choices regarding which hospital they go to. From the perspective of the participants, this leads to the misguiding of the patient, as the majority of patients in Switzerland currently have barely any choice regarding which hospital they go to. Moreover, it is assumed that the published data would not be understood and most ordinary people would be overwhelmed by it.

#### Risk of data manipulation

From the perspective of the participants, self-collected data can be manipulated and distorted by diverse inquiry methods. Knowing that the information will be used for public comparisons, it may be tempting to gloss it over, or to include only those patients with a minimal risk for bad outcomes. In addition, physicians are aware that the coding of the data for medical statistics, which they collect, has to be critically considered.

#### One-sided focus on measurable quality-related variables

The participants felt there was a risk that the so called soft skills such as empathy and having a holistic view of the patient, would be excluded. They were concerned that there would be instead be a one-sided focus on the measurable aspects of quality of care.

"“All these fragmentations in quality measurements, indicators, etc., divide the patient into parts. One forgets that the patient wants to be perceived as a whole. The quality measures to which we also sometimes subject ourselves to, make us to forget what the patient, to whom we should care about, actually wants”."

Another risk is that the other important but less easily measurable aspects of quality could be neglected.

#### Competition prevents co-operation

The targeted competition resulting from publication is judged very negatively among some physicians. It was believed that competition prevented meaningful cooperation among the hospitals.

"“Competition works against any sense of cooperation. For me this is in principle nonsense. It is a mistake to say that competition is of the upmost of importance or that things will then get better. I believe that things will instead get worse, because one no longer cooperates. One becomes a loner”."

Enticement of patients or performance of certain tests in order to bring patients to a hospital, (under the disguise of cost-efficiency) cannot be a meaningful development. For the nurses, the term competition is viewed negatively, as it constitutes a threat to the existing solidarity and collegiality within the profession.

#### Misuse of data

The risk of abuse or misuse of published data is closely related to the role of the media. It is assumed that the media’s priority is to have good headlines with high sales and ratings, and that the conveying of information is not in the foreground. The dominant position the media has and therefore the great influence this gives them with politicians and as well as with public opinion, is seen as problematic. Swiss physicians voiced that they felt a degree of powerlessness against the media. They also stated that as soon as they mentioned the complexity of quality data, it was considered that they were trying to hide something.

## Discussion

The results reveal that the two professional groups have a very critical and negative attitude regarding the publication of quality data in Switzerland. Their views were dominated by the following risks: the reduction of complexity, the one-sided focus on measurable quality variables, risk selection, the threat of data manipulation and the abuse of published information by the media. Unintended negative consequences were also described by Powell et al. In a qualitative study, hospital staff of varying capacities also gave a very critical assessment. They felt that a strong, one-sided focus on the measurable aspects of quality might provoke false incentives for patient care, and could negatively influence provision of care to the patient [19].

The critical attitude is associated with the different value system and the professional self-concept that both physicians and nurses have, in comparison to the underlying principles of a market-based economy. To look at disease and suffering from an economic perspective would be a contradiction. The important professional values such as humanity, caring, empathy and professional autonomy, would be threatened. These values are also essential aspects in relation to career motivation.

The publication of quality data which is accomplished via the reduction of complex issues to individual indicators does not adequately represent the quality of the care among the professions. This would be perceived as discrediting to their work. The previously mentioned values along with the feeling of being discredited, may affect work motivation and identification with the place of employment. These are some of the potential undesirable effects that need to be considered in regards to the publication of quality data. The impending shortage of physicians and nurses must not go placidly ignored among hospital management.

The participants’ criticism regarding complexity reduction is also mentioned in the literature.

This sometimes very distinct reduction signals to society that the illustration and comparison of quality is easily possible. Mason & Street recommend in their article to resist the lure of “over-simplification”. The reductionist approach is contrary to the complexity of hospital operations, and Mason & Street are convinced of and warn against the demotivating and counter-productive messages it gives to hospital employees and to society [5]. The human and financial resources that are required in order to publish quality data should also be taken into consideration. Only marginally reliable findings can be found regarding whether the expected effects actually occur with quality of care and patient safety [3]. The investment of resources in interventions with unclear effects must, in the light of increasingly scarce resources, be critically questioned.

The target of fostering competition in the Swiss health care system via the publication of quality data is viewed by both professions as being an obstacle to efficient and effective health care. Competition inhibits the cooperation of regional hospitals and centers, as well as of specialists. How, and whether or not a competitive environment affects the health care system, is discussed controversially in the literature. Mukamel et.al studied the evolution of the market shares of hospitals after the publication of mortality rates following heart surgery. Hospitals with low mortality rates after surgery registered a higher market share, although this effect only lasted briefly [20]. However, Baker et.al and Chassin found no significant changes in the market shares after the publication of mortality rates [21,22].

Some studies indicate that the publication of quality data increases the quality improvement activities in hospitals. It is, however, unclear as to what extent this effect actually improves treatment results [3]. Such activity increases were observed especially in institutions that had received negative reviews. It is also worth noting that hospitals with poor quality ratings were more likely to engage in improvement activities if these results were openly published, compared to those that received only internal reports or anything at all [23,24].

Despite their critical stance, reflection is required of the two professional groups in order to maintain their unspoken expectation to remain free of public scrutiny and criticism. The demand for transparency of quality performance is legitimate and absolutely justified given the high resource consumption of the health care system. Lack of transparency does not create trust; instead it nurtures speculation that something will be kept secret. Both professional groups are obligated to adequately respond to the legitimate demands of an informed society. This means taking responsibility for the development of appropriate concepts and quality indicators for the publication of quality data. Due to the rather defensive attitude of Swiss health professionals, in particular the medical profession, the financers, the politicians and the media have now filled the vacuum created by their own rather undeveloped concepts that they have proposed and implemented.

### Limitations of the study

The results must be considered in light of some limitations. Due to the qualitative approach, the results cannot be generalized. Theoretical sampling allowed limited insight into the perspectives and attitudes of physicians and nurses. The selection of the hospitals and the composition of the sample were chosen for reasons of practicality, and were neither random nor independent. The participants of the focus groups tended to be very interested and critical regarding the topic. Therefore, possible effects of selection biases regarding the study results cannot be excluded.

Additionally, only a small number of focus groups with limited heterogeneity could be conducted due to limited resources. For example, junior physicians were absent in the physician groups. The discussion, especially those of nurses, was based in part on hypothetical estimates, since they were less able to draw on real experiences. To stimulate discussion, hypothetical scenarios were used with the indicators of mortality, pressure ulcers and patient satisfaction rates. The participants were called upon to present possible outcomes and to formulate their opinions. The focus groups clearly rejected mortality rates as a quality indicator. Whether or not the professionals would have had a different attitude regarding the publication of quality data if they had developed and chosen the indicators by themselves cannot be excluded. Despite a structured analysis process, it cannot be ruled out that the experience and background of the primary author along with her personal opinion regarding the transparency of quality data, may have influenced the results.

## Conclusions

With the release of quality data, the expected negative influences regarding work motivation and the work environment need to be weighed and measured against the possible benefits. It is also of interest to investigate whether or not, or to what extent, the feared data manipulation would appear. Furthermore, evidence that the publication of quality data improves the quality of care is only marginal and is not clearly proven.

Our results can be used as a basis for quantitative assessment of further hypotheses. The effects of the described reactions on Swiss physicians and nurses regarding attitudes on work motivation and identification with their organizations should not be neglected. Due to increasing economic pressures, they have a dilemma between the economical aspects and their humanistic, caring values. These values are those that are most relevant for the motivation and the sense of professionalism. Whenever possible, such factors that threaten these values should be minimized.

## Competing interests

The authors declare that they have no competing interests.

## Authors' contributions

RH is responsible for the conception, design, analysis and interpretation of data. DLBS supervised the conception, design, methods, analysis and interpretation of data. RH drafted the article and DLBS critically revised it. RH and DLBS approved the final version. Both authors had full access to the data and take full responsibility for the integrity of the data and the accuracy of the data analysis. All authors read and approved the final manuscript.

## Pre-publication history

The pre-publication history for this paper can be accessed here:

http://www.biomedcentral.com/1472-6963/12/368/prepub
